# The Regulation of Exosome Generation and Function in Physiological and Pathological Processes

**DOI:** 10.3390/ijms25010255

**Published:** 2023-12-23

**Authors:** Ying Wang, Tong Xiao, Chaoran Zhao, Guiying Li

**Affiliations:** Key Laboratory for Molecular Enzymology and Engineering of the Ministry of Education, School of Life Sciences, Jilin University, Changchun 130012, China; wying21@mails.jlu.edu.cn (Y.W.); xiaotong1320@mails.jlu.edu.cn (T.X.); zhaocr1321@mails.jlu.edu.cn (C.Z.)

**Keywords:** extracellular vesicles, exosome, exosome biogenesis, cargo sorting, secretion, tumor-derived exosomes

## Abstract

Exosomes, a type of extracellular vesicle with a diameter of approximately 100 nm that is secreted by all cells, regulate the phenotype and function of recipient cells by carrying molecules such as proteins, nucleic acids, and lipids and are important mediators of intercellular communication. Exosomes are involved in various physiological and pathological processes such as immunomodulation, angiogenesis, tumorigenesis, metastasis, and chemoresistance. Due to their excellent properties, exosomes have shown their potential application in the clinical diagnosis and treatment of disease. The functions of exosomes depend on their biogenesis, uptake, and composition. Thus, a deeper understanding of these processes and regulatory mechanisms can help to find new targets for disease diagnosis and therapy. Therefore, this review summarizes and integrates the recent advances in the regulatory mechanisms of the entire biological process of exosomes, starting from the formation of early-sorting endosomes (ESCs) by plasma membrane invagination to the release of exosomes by fusion of multivesicular bodies (MVBs) with the plasma membrane, as well as the regulatory process of the interactions between exosomes and recipient cells. We also describe and discuss the regulatory mechanisms of exosome production in tumor cells and the potential of exosomes used in cancer diagnosis and therapy.

## 1. Introduction

Exosomes are a type of extracellular vesicle (EV) derived from endosomes, serving as carriers for intercellular signal transmission [1]. EVs are membranous vesicles secreted by cells into the extracellular environment and functionally mediate intercellular communication and regulate the biological activity of recipient cells through their carrying proteins, nucleic acids, lipids, etc. [2]. Given its important role in mediating communications between cells, research on EVs has become a major field of cell biology. In the last decade, extracellular vesicles (EVs) have become a rapidly growing focus of biomedical research as they play new roles in physiological and pathological regulation, showing high potential as biomarkers and clinical applications [2]. Based on the different processes of formation, EVs can be broadly classified into two categories: ectosomes and exosomes. Ectosomes, including microvesicles, microparticles, and macrovesicles, with diameters ranging from approximately 50 nm to 1000 nm, are entrapped on the surface of the plasma membrane by direct outward budding and subsequently released into the extracellular space. By contrast, exosomes originate from ILVs (intraluminal vesicles) that bud inward through the limiting membranes of mature endosomes, and these ILVs range from approximately 40 nm to 160 nm (average ~100 nm) in diameter [2]. Endosomes with intraluminal vesicles are often referred to as multivesicular endosomes (MVEs) or multivesicular bodies (MVBs) [3]. Exosomes, as specific secreted membranous vesicles, are involved in various physiological and pathological processes such as immunomodulation, immunotherapy, immune response, promotion of matrix remodeling, and angiogenesis. In addition, exosomes regulate tumorigenesis and development by promoting tumor cell proliferation, increasing chemoresistance and enhancing cell migration [4]. Numerous studies have shown that exosomes have become an important biomarker for clinical diagnosis, and their in-depth study can help to reveal the physiopathological mechanisms, while their potential application in diagnosis and treatment has a broad prospect [5].

We already know that almost all cells produce exosomes, and tumor cells produce more exosomes than healthy cells [6]. These tumor-derived exosomes act as intercellular mediators to achieve communication with cancer cells themselves and other cells in an autocrine and paracrine manner, thereby regulating the activity of these cells, such as tumorigenesis, invasion, and metastasis. The regulatory activity of exosomes depends on the secretion and uptake of exosomes, as well as the composition of their vesicular inclusions. However, knowledge of the molecular mechanisms underlying exosome biogenesis, composition, secretion, and corresponding functions is still very limited. Here, we will discuss the reported regulatory mechanisms of each process in the endosomal pathway that affects the biogenesis of exosomes, including endocytosis, cargo sorting, MVB formation, secretion, and the regulatory mechanisms of uptake for the interaction between exosomes and recipient cells, as well as how these processes participate in the regulation of exosome production in cancer cells.

## 2. The Biogenesis and Secretion of Exosomes

**Figure 1:**
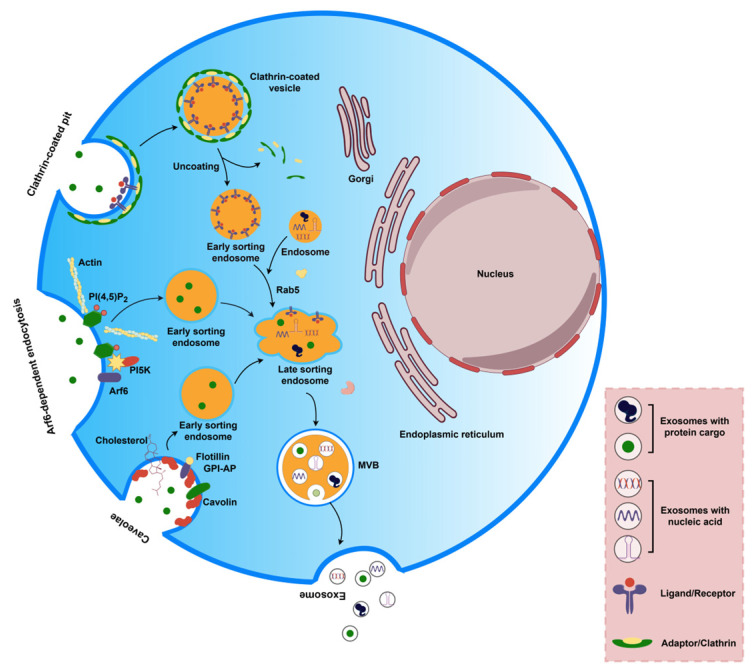
The process of exosome formation. Firstly, the cytoplasmic membrane invaginates, wrapping the extracellular components and cytosolic proteins together to form early-sorting endosomes (ESEs). The formation of ESEs consists of two pathways: clathrin-mediated endocytosis (CME) and clathrin-independent endocytosis (CIE). CME is mediated by a variety of molecules, including clathrin proteins, constriction, and release processes, and eventually, clathrin proteins are shed to form ESEs. CIE takes two forms: the caveola-dependent endocytosis pathway, where plasma membranes are trapped in lipid rafts rich in molecules such as GPI-anchored proteins, cholesterol, and other molecules to form ESEs under the guidance of caveolins and flotillin, and the Arf6-dependent endocytosis pathway, where Arf6 activates phosphatidylinositol kinase PI5K to produce plasma membrane phosphatidylinositol PI(4,5)P_2_, which in turn drives endocytosis by recruiting microfilament assemblies to form ESEs. Then, the ESEs produced through different pathways, with the involvement of proteins such as Rab5 to form late-sorting endosomes (LSEs), and late endosomal membranes bud inward to form intracellular multivesicular bodies (MVBs), which contain many intraluminal vesicles (future exosomes), and eventually MVBs fuse with the cell plasma membrane to release exosomes outside. (By Figdraw. Hangzhou, Duotai. Technology Co., Ltd., Hangzhou 310000, China).

The biogenesis of exosomes is related to the maturation pathway of endosomes [7]. The production process of exosomes involves the first invagination of the plasma membrane to form a cup-like structure encapsulating cell surface proteins and extracellular components, leading to the formation of early-sorting endosomes (ESEs). And then, these ESEs can mature through acidification and exchange substances with other organelles to form late-sorting endosomes (LSEs). Next, the membrane of the endosome is inwardly budding (i.e., double invagination of the plasma membrane) to form intracellular multivesicular bodies (MVBs), which will contain many intraluminal vesicles (ILVs). Finally, ILVs may be released to become exosomes [2] (Figure 1). Exosome production and release can be inhibited or facilitated by internal cellular processes or external stimuli and are regulated by a variety of molecular mechanisms [8].

### 2.1. Formation of ESEs for Exosome Biogenesis

#### 2.1.1. Mechanisms of ESE Formation

The biogenesis of exosomes is intensely regulated. Helenius et al. [9] showed that exosome biogenesis began with the ESE. The ESE can consist of a variety of receptors (cell signaling receptors, nutrient transporter proteins, ion channels, adhesion molecules, and polarity markers), lipid membranes, and extracellular fluid. ESE is an early large vesicle formed by the fusion of primary endocytic vesicles [9]. Currently, there are two main explanations for the formation of endocytic vesicles: clathrin-mediated endocytosis (CME) and clathrin-independent endocytosis (CIE) [10].

CME is characterized by the involvement of clathrin, which is a triskelion consisting of three heavy chains and three light chains [11]. Through adapter protein complexes, clathrin encapsulates various cargo proteins that require internalization, forming clathrin-coated pits (CCPs) and clathrin-coated vesicles (CCVs). Adaptor proteins usually bind to short peptides in the intracellular domains of transmembrane proteins, while some bind to phosphatidylinositol PI(4,5)P_2_, which is abundant in the plasma membrane, and some adaptor proteins can bind to ubiquitin [12]. Adaptor proteins such as AP2 and other adaptor proteins such as DAB-1 found in *Caenorhabditis elegans* mediate cargo recognition and CCP assembly, followed by rapid recruitment of clathrin [13], through their intramolecular isoleucine zip domains such as the XXXL (L/I/M) [14] and PTB (phosphotyrosine-binding) domains [15]. During this process, BAR (Bin-Amphiphysin-Rvs) structural domain arcuate proteins, such as endophilin and sorting nexin 9 (SNX9), promote membrane curvature deformation while aggregation of clathrin further stabilizes the invagination morphology of the CCP and facilitates the maturation of the CCV [16,17]. The scission and release of mature CCVs from the plasma membrane is dependent on dynamin, a GTPase protein that is recruited to the CCP by interacting with endophilin and the SRC homology 3 (SH3) domain of SNX9 via a proline-rich domain (PRD) [18]. Dynamin surrounds and assembles into a neck-ring structure at the junction of the deeply invaginated CCV and the plasma membrane, which converts chemical energy into mechanical energy to drive membrane scission through GTP hydrolysis. After the scission of the CCV from the plasma membrane, the clathrin coat is shed by the combined action of ATPase, heat shock homolog 70 (Hsc70), and its cofactor auxilin [19].

CIE is a collective term for various clathrin-independent endocytosis pathways, which mainly consist of caveola-dependent endocytosis, CLIC/GEEC (clathrin-independent carriers/GPl-enriched endocytic compartments), and small GTPase Arf6-dependent pathway [20]. Caveola-dependent endocytosis mediates lipid raft-associated cargo endocytosis by the major structural proteins of caveola, caveolin-1, 2, and 3. The caveola is formed in lipid raft regions enriched in cholesterol, sphingolipids, and glycosylphosphatidylinositol (GPI)-anchoring proteins. The membrane-associated proteins AnxA2 and flotillin are components of lipid rafts and positively regulate lipid raft-mediated endocytosis. Both clathrin- and caveolin-mediated endocytosis involve dynamin molecules. The CLIC/GEEC pathway is not dynamin-dependent but is associated with the Rho family small G proteins RAC1 and CDC42. This pathway is associated with the formation of microfilament-dependent and clathrin-independent carriers [21]. Via the CLIC/GEEC pathway, endocytic vesicles fuse to form phosphatidylinositol-enriched early endosomes [20]. The Arf6-dependent pathway mainly mediates the uptake and recycling of histocompatibility antigen I [22]. It mainly regulates the assembly of coating proteins, participates in the vesicle budding process, and is essential for cargo sorting after endocytosis [23]. Arf6 is localized on the cell membrane where Arf6 activates the phosphatidylinositol-5-kinase (PI5K) to produce the plasma membrane PI(4,5)P_2_, which in turn may drive endocytosis by recruiting microfilament assembly [24]. In fact, cargo proteins that enter cells via the CIE pathway are crucial for cell survival, signal transduction, cell migration, and regulation of small G-protein activity. Therefore, the cyclic transport of CIE cargo membrane proteins has also been implicated in the occurrence and development of human diseases, especially cancer [20] (Figure 1).

In addition, immune cells such as macrophages and dendritic cells employ phagocytosis to internalize small particles such as exosomes. Phagocytosis is dependent on phosphatidylinositol-3-kinase (PI3K) and actin cytoskeletal activity, where the cell membrane deforms to enclose a large number of extracellular particles, forming phagosomes that ultimately direct internalized cargo to lysosomes. PI3K and phospholipase C (PLC) are required for phagosome closure [25].

The different endocytosis mentioned above can co-exist, e.g., ovarian cancer and melanoma cells predominantly utilize a cholesterol-associated lipid raft endocytosis mechanism, but there is also concomitant clathrin-mediated endocytosis, phagocytosis, and micropinocytosis [26].

#### 2.1.2. The Regulation of Rab in Endocytosis

Rab small GTPases represent the most extensive subfamily within the Ras small G-protein superfamily. These proteins undergo a transition between active and inactive states via GTP- and GDP-bound forms [27]. The GTP-bound Rab selectively binds to effectors implicated in regulating vesicle formation, transportation, and fusion [28]. Rab5 is a major regulator of endocytic vesicles. Active Rab5 recruits a series of effectors to early endosomes [29], facilitating the fusion of newly formed endocytic vesicles with early endosomes, as well as the homotypic fusion of early endosomes. Rabs5 (Rabenosyn5), the Vps45 protein (vacuolar protein sorting 45), and Vps34 are effectors of Rab5 that participate in the fusion process of endosomes in *Caenorhabditis elegans*. Vps34 is classified as a type III phosphatidylinositol kinase, which is known to exert a major impact on the processes of autophagy, endosome trafficking, and phagocytosis. Vps34 facilitates the synthesis of phosphatidylinositol PI(3)P on early endosomes. Vps34 is able to bind and recruit a variety of early endosomal membrane proteins, affecting endosome fusion or facilitating subsequent recycling and degradation processes [30,31].

The process of converting from early endosomes to late endosomes involves the Rab cascade reaction [32]. The upstream Rab (Rab5) recruits the GMP exchange factor (GEF) of the downstream Rab (Rab7), which activates the downstream Rab. In turn, the activated downstream Rab recruits the GTPase-activating protein (GAP) of the upstream Rab, and the guanylate transferase activity of GAP shuts down the activity of the upstream Rab. In this way, the Rab cascade is able to transform the molecular composition of the membrane and thus affect the properties and functions of the endosome. Ultimately, Rab5-labeled early endosomes are transformed into Rab7-labeled late endosomes.

### 2.2. The Formation of Intraluminal Vesicles (ILVs)

Following the process of ESE formation, the endosomal membrane initiates a budding process toward the lumen, resulting in the generation of numerous small intraluminal vesicles (ILVs). Over time, these ILVs accumulate and give rise to multivesicular bodies (MVBs), signifying the maturity of endosomes. MVBs are a class of late endosomes that contain numerous ILVs (future exosomes) in the lumen [33]. The formation of ILVs is accompanied by membrane remodeling processes such as enrichment and sorting of cargo molecules on the endosome membrane, membrane invagination, and budding. MVBs can undergo fusion with lysosomes, leading to the degradation of intraluminal vesicles (ILVs), which plays a crucial role in the regulation of various biological processes, including nutrition absorption, immune responses, and cellular signaling transmission [34]. Also, MVBs can undergo fusion with the plasma membrane in order to release ILVs, which serve as exosomes facilitating intercellular communication [35]. MVBs are highly heterogeneous. Organisms possess a variety of complex and diverse regulatory mechanisms to maintain the diversity of MVBs [36].

The currently known regulators of MVBs production mainly include endosomal sorting complexes required for transport (ESCRT), specific lipids (ceramide, phosphatidic acid, sphingosine-1-phosphate), and tetraspanning family proteins. Among them, the ESCRT-dependent mechanism for the inward budding of the endosomal membrane has been described in detail in the literature [37] and will not be highlighted in this review.

#### 2.2.1. Effect of Lipids on the Production of MVBs

The physiological properties of lipids are critical to cell membrane remodeling. Exosome membranes are rich in sphingolipids, glycerophospholipids, ceramides, and cholesterol [38], which highlights that specific lipid components may contribute to the MVB sorting and formation process. Studies have shown that the cholesterol content of endosomal membranes may affect the fate of the generated exosomes. Cholesterol-rich MVBs tend to secrete exosomes extracellularly, but low cholesterol levels make MVBs prone to lysosomal degradation [33,39].

Sphingomyelin ceramide is thought to play an important role in the formation of EVs. First, ceramide can promote the formation and further expansion of sphingomyelin-rich lipid raft microdomains on the endosomal membrane, thereby inducing the budding of the endosomal membrane. Secondly, the smaller hydrophilic head and larger hydrophobic tail of lipid molecules such as ceramide and phosphatidic acid are similar in structure to a pyramid, so when they are embedded in the endosomal membrane, they can induce the membrane to indent, thereby promoting the formation of ILVs. Ceramide can also be metabolized to produce sphingosine 1-phosphate (S1P), which binds to the S1P receptor on MVBs. The activated S1P receptor promotes the polymerization of microfilaments on endosomal membranes by activating the small G proteins CDC42 and Rac1, which in turn facilitates cargo sorting to ILVs [40,41,42,43]. This process is considered independent of ESCRT.

The production of ceramide is regulated by neutral sphingomyelinase-2 (nSMase2). By decomposing sphingomyelin (SM), nSMase2 increases the levels of ceramide in the plasma membrane and/or endosomal membrane system. Other enzymes in sphingolipid metabolism, such as sphingomyelin synthase (SMS)1 and 2, have also been shown to regulate extracellular vesicle formation. They convert ceramide to SM at the Golgi and plasma membrane, respectively [44]. A recent study showed that ceramide transfer protein (CERT) was involved in regulating the sphingolipid composition of EVs. CERT recognizes and forms a complex with the Tsg101 subunit of ESCRT-I in MVBs and mediates ceramide in the direct transport between the endoplasmic reticulum and late endosome MVBs, determining the ceramide content of EVs, which links ceramide to ESCRT-dependent pathways.

#### 2.2.2. Effect of Tetraspanin on the Production of MVBs

Another ESCRT-independent pathway for exosome cargo sorting and production is related to the tetraspanins (CD81, CD10, CD9, CD63). The tetraspanins, which are a protein superfamily, play a key part in organizing membrane microregions through the formation of clusters and interactions with various transmembrane and cytoplasmic signaling proteins. These interactions result in the generation of tetraspanin-enriched microdomains (TEMs), which function as platforms for the transport of cargo [45]. As an illustration, it has been observed that the melanocyte-specific glycoprotein PMEL (pre-melanosomal protein) undergoes entrance into ILVs following its interaction with CD63 [46]. Similarly, the membrane metalloprotease CD10 engages with CD9 to facilitate its entry into ILVs. Additionally, the proteins CD9 and CD82 connect with E-cadherin to enhance the exocytosis of β-catenins that are conducted via exosomes [46,47]. Moreover, these supramolecular complexes in the cell membrane not only regulate the transport of exosomal cargoes but also influence exosome production. A previous investigation demonstrated that the depletion of CD9 resulted in a decrease in exosome secretion from dendritic cells in mice [48]. A separate study showed that the tetraspanins CD81 and CD82 play a role in controlling the development of cell membrane protrusions, suggesting their involvement in the regulation of exosome budding [49]. However, the mechanism by which tetraspanins regulate exosome production is not fully understood. According to a recent study, it was observed that the upregulation of CD63 inhibits its own endocytosis, thereby initiating the formation of CD63 exosomes. Additionally, the knockout of CD63 expression by CRISPR/Cas9 resulted in a decrease in exosome secretion but did not affect the formation of ectosomes. These findings suggest that CD63 contributes to the biogenesis of exosomes [50].

### 2.3. Exosome Cargo Sorting

The contents of exosomes consist mainly of nucleic acids, proteins, and lipids, which play a key role in the process of intercellular communication. The mechanism of cargo sorting has been reported in a large number of studies [37], and here, we briefly add a description of the sorting of nucleic acid cargoes and nucleo-cytoplasmic transport-protein-mediated sorting of exosome cargoes.

#### 2.3.1. Nucleic Acid Cargo Sorting

In addition to protein cargoes, exosomes are enriched with heterogeneous RNA cargoes. These RNA cargoes include mRNAs, miRNAs, and other non-coding RNAs such as ribosomal RNAs (rRNAs), transfer RNAs (tRNAs), Y-RNAs, and long intergenic non-coding RNAs (lincRNAs) [51,52]. Directed entry of RNA into exosomes involves highly efficient sorting mechanisms, which allow for some specific RNAs to be loaded into exosomes while other RNAs are barely detectable in exosomes. Among them, miRNA’s exosome sorting mechanism has attracted much attention due to its important cellular regulatory functions [53].

Several pathways and molecules have been reported for exosomal miRNA sorting, including lipid/ceramide, miRNA modification, and interactions with components of the MVB biogenesis pathway. For example, nSMase2, a key enzyme in the sphingolipid-ceramide metabolic pathway, regulates miR-210 levels in exosomes derived from cancer cells [54] and MSCs [55] by hydrolyzing SM to produce ceramides. nSMase2 also regulates the entry of miR-10b into exosomes derived from metastatic breast cancer cells [56]. In addition, recent evidence suggests that modification of the 3′ end of miRNAs, methylation, etc., may also affect their selective exosome packaging [57,58]. In addition, SUMO (small ubiquitin-associated modifier) modification of heterogeneous nuclear ribonucleoprotein hnRNPA2B1 in exosomes regulates the binding of hnRNPA2B1 to miRNAs, thereby triggering sorting [57].

RNA-binding proteins (RBPs) are key players in post-transcriptional processing and RNA molecular regulation. Their direct interactions with MVB components are associated with exosomal miRNA sorting. Many RBPs, such as heterogeneous nuclear ribonucleoprotein (hnRNP), YBX1, MVP, MEX3C, SYNCRIP, argonaute 2 (Ago2), and FMR1, are involved in ILVs/exosomes sorting of miRNAs in different cells [59]. However, the mechanism by which MVBs recognize RBP-RNA complexes and how RNA is sorted in exosomes remains poorly understood. Among them, hnRNP is a protein superfamily consisting of more than 20 proteins, and certain members of the family are involved in miRNA sorting. In addition to hnRNPA2B1, hnRNPA1 can recognize and bind miRNA-specific motifs (EXO motifs) to mediate exosomal miRNA sorting. For example, hnRNPA1 can mediate miR-196a sorting in cancer-associated fibroblast (CAF)-derived exosomes by binding to a specific motif (UAGGUA) at the 5′ end of miR-196a [60]. hnRNPA1 can also recognize a specific motif (UAGGUA/AGAGGG) in leukemia cells to sort miR-320 in exosomes [60].

Other protein components in MVBs have also been associated with miRNA sorting. It has been shown that in human hepatic stem cell-like cells, the adapter protein Alix regulates the RNA loading of EVs [61]. Alix recruits the RNA-binding protein Ago 2 to the endosomal membrane, which in turn induces miRNA binding and subsequent packaging into EVs. Moreover, Ago2 levels can affect let-7a, miR-100, and miR-320a sorting. In addition, the autophagy marker microtubule-associated protein 1 light chain 3β (LC3B) has been detected in exosomes. LC3B has been shown to bind to RNA-binding proteins such as hnRNPK and scaffold attachment factor B (SAFB) to mediate miRNA cargo sorting [62]. Serine/arginine-rich splicing factor 1 (SRSF1) can recognize and bind to the specific motif of miR-1246 to make it highly enriched in the exosomes derived from cancer cells, especially pancreatic cancer cells [63,64]. In recent years, some research work has found that Y box binding protein-1 (YBX-1) is involved in miRNA sorting, but the explicit mechanism deserves further investigation [65].

#### 2.3.2. Nucleo-Cytoplasmic Transport-Protein-Mediated Sorting of Exosome Cargoes

Recent studies have shown that nucleo-cytoplasmic transport (NCT)-regulating proteins are involved in the sorting of exosome cargoes [66]. Ran, as a small GTPase, is a highly conserved protein required for the NCT of macromolecules through the nuclear envelope (NE) [67,68]. The asymmetric distribution of Ran GTPase-activating protein 1 (RanGAP1) in the cytoplasm and guanine nucleotide exchange factor RCC1 in the nucleus results in a steep gradient of RanGTP inside and outside the NE, where RanGTP is more concentrated in the nucleus, whereas RanGDP is mainly in the cytoplasm. This gradient determines the directionality of transport, allowing the import complex to assemble in the cytoplasm and disassemble in the nucleus with the participation of importins, while the export complex assembles in the nucleus and disassembles in the cytoplasm with the participation of exportin 1 (XPO1), also known as chromosome region maintenance 1 (CRM1) [66,68]. Some RanGTP-CRM1-NES (nuclear export signal) cargo export complexes may escape catabolism in the cytoplasm and be recruited to MVBs produced by the inward budding of late endosomal membranes. The MVBs eventually fuse with the plasma membrane and release the luminal vesicles. In recipient cells, this complex may be disassembled, thereby releasing cargo due to RanGAP1-mediated hydrolysis of GTP on Ran in the cytoplasm. This implies that the NCT mechanism plays a role in sorting a portion of the cargo into exosomes released by the donor cells [69]. Chavan et al. [69] have found that the key NCT protein, RanGTP, is enriched in exosomes secreted by mammalian cells. The loading of the soluble protein GAPDH into the EVs (including exosomes) is also closely associated with the Ran/CRM1 nuclear translocation complex. Furthermore, Ran and CRM1 are overexpressed in a variety of cancers [66,70], and exosome production is often enhanced in cancer cells [71], so it is important to further investigate the link between NCT complexes and EVs.

CD47 is an inhibitory receptor expressed on the surface of normal cells and tumor cells. CD47, as the receptor of thrombospondin-1 (TSP1), is involved in the regulation of antiangiogenesis [72]. The interaction of CD47 with signal-regulating protein α (SIRPα) produces “don’t eat me” signals [73]. CD47 was identified as a component of extracellular vesicles released by various cell types and one of 22 universally enriched proteins in exosomes [74]. Kaur et al. found that CD47 expression regulates the RNA composition of released EVs, and T-cell exosomes can regulate VEGF signaling of recipient cells in a CD47-dependent manner [75]. Recently, their results have indicated that the interactions of CD47 with components of the Ran/XPO1 nuclear export complex via ubiquilin-1 limit the nuclear export and EV enrichment of some cargo proteins and RNAs [76]. Exportin-1 regulates the transport of 5′-7-methylguanosine (m^7^G)-modified microRNAs and mRNAs from the nucleus to the cytoplasm [77]. Kaur et al. [75] found that covalent modification of XPO1 using the highly specific inhibitor LMB (leptomycin B) resulted in the inactivation of XPO1, which inhibited the export of cargo proteins and m^7^G-capped RNAs from the nucleus. LMB also inhibited the binding of XPO1 to CD47 and increased the levels of m^7^G-modified RNAs in released EVs. Although the mechanisms by which exportin-1 and CD47 regulate RNA packaging into EVs directly or via MVBs remain to be further investigated, this study demonstrates the importance of nucleo-cytoplasmic transport proteins in regulating EV release and sorting of EV cargoes.

### 2.4. Transport of MVBs

**Figure 2:**
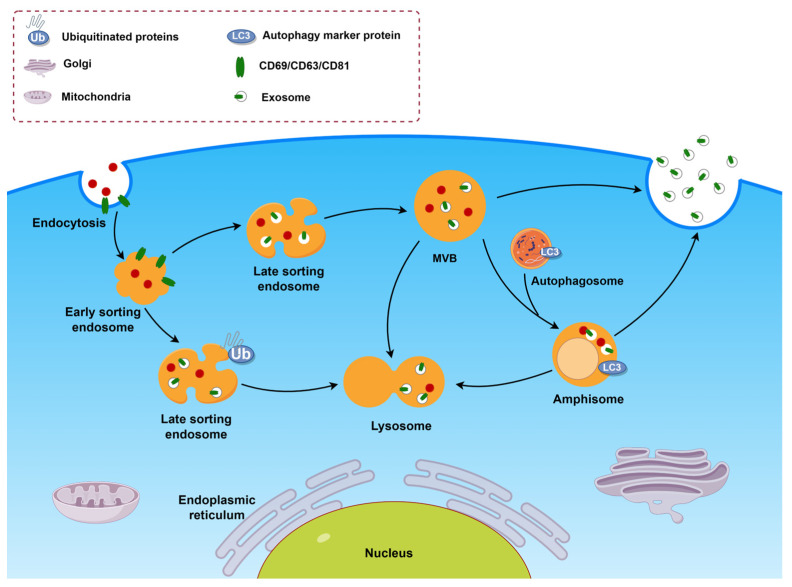
The fate of mature MVBs. Mature MVBs face three “fates”: MVBs fuse with lysosomes to transport ubiquitination-modified proteins to lysosomes for degradation and recycling; MVBs fuse with the plasma membrane to secret exosomes; MVBs can also fuse with autophagosomes to form amphisomes, which can both fuse with lysosomes to be degraded and with the plasma membrane to secrete exosomes. (By Figdraw. Hangzhou, Duotai. Technology Co., Ltd., Hangzhou 310000, China).

After their formation, MVBs face three “fates” [78] (Figure 2). One is responsible for delivering cargo molecules from the endocytosis pathway to lysosomes for degradation and recycling, regulating biological processes such as nutrient uptake, immunity, and signal transduction. MVBs can also fuse with the cell plasma membrane to release ILVs, which play an intercellular communication role in the form of exosomes. In addition, MVBs can also fuse with autophagosomes to form amphisomes, which can fuse with lysosomes to be degraded or fuse with the plasma membrane to secrete exosomes. Under physiological conditions in normal cells, there is usually a dynamic balance between the production of MVBs, fusion with lysosomes or autophagosomes for degradation, and the release of exosomes [79]. However, under pathological conditions, tumor tissue secretes more exosomes and promotes the occurrence and development of tumors by altering the tumor microenvironment, angiogenesis, and modifying the body’s immune system [80]. Therefore, the study of the fate and regulatory mechanisms of MVBs has important physiological and clinical significance.

#### 2.4.1. Regulation of MVBs Fusion with Lysosome

The fusion of MVBs with lysosomes is regulated by various proteins and mechanisms. For example, tetraspanin 6 (TSPAN6) is involved in the regulation that occurs between lysosomal degradation and exosome secretion. TSPAN 6 is enriched in MVBs and ILVs, recruiting the cytoplasmic adapter protein syntenin through its PDZ1 domain, thereby promoting MVBs-plasma membrane fusion. At the same time, TSPAN 6 enrichment reduced the fusion between MBVs and lysosomes and increased the release of exosomes [81]. Post-translational ubiquitin-like modification, ISGylation, is one of the regulatory mechanisms for the fate of MVBs [82]. The ubiquitin-like modification of MVB proteins mediates the MVBs fusion with lysosomes and degradation [83]. The ubiquitination modification of polymerase I and transcription release factor (PTRF, also known as caveolin-associated protein-1, CAVIN1) plays an important role in the formation of small pits and the secretion of exosomes. PTRF in exosomes has been identified as a potential biomarker for various malignant tumors such as glioma and renal cell carcinoma. A study shows that the ubiquitin-binding enzyme E2O (UBE2O) downregulates the release of exosomes through the ubiquitination of PTRF to regulate the secretion of exosome-related PTRF [84].

#### 2.4.2. Regulation of Fusion between MVBs and Plasma Membrane

- Actin cytoskeleton and MVB transport

The transport of MVBs to the plasma membrane is a prerequisite for exosome release, in which the actin cytoskeleton plays an important role [85]. Furthermore, diverse types of microfilament regulators are involved in regulating endosomal recycling transport. In *C. elegans*, the small G protein Rab10 can regulate microfilament bundling on vesicles through its effector microfilament bundling protein EHBP-1 [86]. The CH domain and C2-like domain of the EHBP-1 can bind to PI(4,5)P_2_ on microfilaments and endosomes, respectively. Meanwhile, the C-terminal CC domain interacts with Rab10 to enhance the affinity between the CH domain and microfilaments. Through the interactions mediated by the different domains mentioned above, EHBP-1 bridges recycling endosomes with the microfilament skeleton to promote recycling [28]. In a study using *C. elegans* as a research model, a new factor regulating the production of MVBs, the filament-binding protein FLN-2, was identified. FLN-2 can directly interact with F-actin and the V1-E subunit VHA-8 of V-ATPase through its N-terminal CH domain, mediating the anchoring of MVB to the microfilament skeleton to promote its recycling. Loss of this function leads to the reduction of MVBs and ILVs [87]. In the early endosomal sorting pathway of exosomes, the uptake and endosomal recycling of histocompatibility antigen I are mediated by Arf6 through PI(4,5)P_2_, recruiting actin regulatory factors to participate in the dynamic regulation of microfilament skeleton [88]. Cortactin is an actin-binding protein that has been confirmed to be overexpressed in a variety of tumors and participates in a variety of biological processes that rely on branched actin filaments, such as cell movement, invasion, and membrane transport [89]. Sinha et al. [90] found that actin can control the movement of MVBs and their implantation on the plasma membrane. Additionally, actin can bind branched actin filaments and actin-related protein complexes (Arp2/3 complex) to regulate exosome secretion. Moreover, overexpression of cortactin in cancer cells can promote the secretion of exosomes.

- Rab regulates the transport of MVBs to the plasma membrane

Rab protein is an important molecular switch protein in the formation, transport, anchoring, and fusion process of vesicles, and its regulatory role in all aspects of vesicle transport is very important. In mammals, approximately 60 different Rabs have been identified, each exhibiting specific intracellular localization and regulating different steps of intracellular membrane transport (Table 1). Rab5, a small GTPase of the Ras family, travels from the plasma membrane to early endosomes and regulates vesicular transport and fusion of the plasma membrane with early endosomes through interactions with other proteins [29]. Wang et al. found that Rab10 enhanced the attachment of EHBP-1 to actin by interacting with its effector EHBP-1, thereby linking the membrane lipid PI(4,5)P_2_ to the actin cytoskeleton to promote endosomal tubularization [86]. Collectively, when Rab27 (Rab27A and Rab27B) is knocked down, it attenuates exosome release. Rab27 has been identified as a direct regulator of MVB transport to the plasma membrane [91]. The Rab7 adapter protein RILP (Rab-interacting lysosomal protein) is critical for connecting Rab7-positive vesicles to the dynein–dynactin complex. RILP is cleaved into fragments (cRILP) following inflammation or viral infection. However, cRILP can not combine with GTPase Rab7 to form a dynein–dynactin complex, which changes the direction of vesicle transport to lysosomes but promotes kinesin-mediated movement towards the cell surface [92,93]. Defects to Rab family members (such as Rab7, Rab11, Rab27A/B, and Rab35) also affect the release of exosomes [94]. For example, in neutrophils [95] and primary oligodendrocytes [96], it has been found that inhibiting Rab35 expression can reduce exosome secretion. Subsequently, it has also been confirmed in human RPE1 cells that exosome secretion requires the participation of Rab11 or Rab35 [97]. Rab11A has been confirmed to regulate the transport of MVBs in head and neck cancer cells [98]. It has been reported that EGFR-phosphorylated Rab31 interacts with flotillin proteins in lipid raft microdomains to induce EGFR entrance into MVEs to generate ILVs independent of the ESCRT mechanism. Active Rab31 binds to the SPFH domain and promotes ILV formation through the flotillin domain of flotillin proteins. Meanwhile, Rab31 engages the GTPase-activating protein TBC1D2B to inactivate Rab7, inhibiting MVE-lysosome fusion and allowing ILVs to be secreted as exosomes [7]. The small GTPase Rab22A colocalizes with budding MVs at the cell surface and coordinate with its effectors and its regulators to allow various types of internalized cargos with either sequence-independent or sequence-dependent sorting motifs to move along distinct recycling pathways [99]. Moreover, Rab22A also plays a crucial role in the MHC I endocytic trafficking, which is vital for efficient cross-presentation by dendritic cells [100].

In addition, recent studies have found that certain lncRNAs can indirectly affect membrane docking and fusion by disturbing the expression and localization of Rab proteins. lncRNA HOTAIR affects the co-localization of VAMP3 and SNAP23 by regulating the expression and localization of Rab35 and ultimately promoting exosome secretion [101]. It has also been found that lncRNA PVT1 promotes the docking of MVBs to the plasma membrane by changing the expression and localization of Rab7 and regulating the co-localization of YKT6 and VAMP3.

- The impact of changes in PIP levels on endosomal recycling

Changes in PIP levels on the endomembrane are also critical for the directional flow of cargos. PIP is a phosphorylated derivative of phosphoinositide (PI), which is synthesized in the endoplasmic reticulum and delivered to other endomembrane compartments via membrane transport [97]. The PI inositol ring can be phosphorylated at three different positions (D3, D4, D5) to form seven possible forms of PIP. Respectively, PI(4)P, PI(4,5)P_2_, PI(3)P, and PI(4,5)P_2_ are mainly enriched in the Golgi apparatus, the plasma membrane, and recycling endosomes, early endosomes, and late endosomes and lysosomes [102].

**Table 1:** Summary of Rab proteins participating in the regulation of exosome biogenesis.

Rab Proteins	Effects	References
Rab5	Recruiting a series of effectors to early endosomes and mediating the fusion of newly formed endocytic vesicles with early endosomes and the homotypic fusion of early endosomes	[29]
Rab7	Involved in the Rab cascade reaction	[92,93]
Rab10	Regulating microfilament bundling on vesicles through its effector microfilament bundling protein EHBP-1	[86]
Rab27A/B	Promoting the targeting of MVEs to the cell periphery and their docking at the plasma membrane	[91]
Rab11A	Promoting docking and fusion of MVBs in a calcium-dependent manner	[98]
Rab35	Switching on OCRL recruitment on newborn endosomes, post-scission PtdIns(4,5)P_2_ hydrolysis, and subsequent endosomal trafficking	[94,95,96,97]
Rab31	Contact with flotillin proteins in lipid raft microdomains drives epidermal growth factor receptors into MVE to form ILV while recruiting the GTPase-activating protein TBC1D2B to inactivate Rab7, thereby preventing MVE fusion with the lysosome and allowing ILV to be secreted as an exosome	[7]
Rab22A	Localizing to early endosomes and participating in sorting and recycling of molecular cargos internalized by clathrin-dependent and -independent mechanisms	[100,103]

#### 2.4.3. Regulation of MVBs Fusion with Autophagosomes

MVBs can also fuse with early autophagosomes to form amphisomes, which then fuse with lysosomes and degrade the contents. The fusion of amphisome and lysosomes, as well as the mutual transport between autophagosomes and late endosomes/lysosomes, require the coordinated action of multiple membrane dynamics regulatory proteins, including SNAREs, tethering proteins, and RabGTPases [64]. Using bafilomycin A1, a selective inhibitor of V-ATPases, to inhibit the autophagy pathway can prevent the fusion of amphisomes and lysosomes, thereby facilitating the release of exosomes. On the other hand, amphisomes can also fuse with the plasma membrane and then release vesicles into the extracellular space [83,104]. For example, in lung epithelial cells, interferon-γ (IFN-γ)-induced autophagy can promote the fusion of annexin A2 (ANXA2)-containing autophagosomes with MVBs and subsequent exosome release [105]. The above indicates that autophagy is involved in the regulation of exosome secretion by affecting the transport of MVBs.

### 2.5. Exosome Secretion

**Figure 3:**
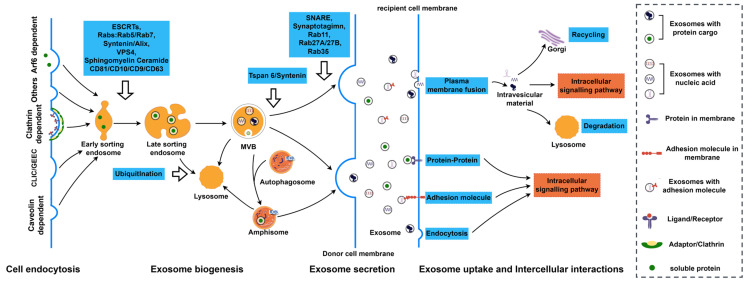
The life of an exosome. The exosomes originate from endocytosis and form early-sorting endosomes. Exosome biogenesis relies on both ESCRT-dependent and ESCRT-independent pathways to sort cargos and form MVBs by budding inward. Some MVBs deliver cargo molecules to lysosomes for degradation and recycling. Some fuse with the plasma membrane to release ILVs, which play a role in intercellular communication in the form of exosomes. Other MVBs fuse with autophagosomes to form amphisomes, which can fuse with lysosomes for degradation, as well as with the plasma membrane to secrete exosomes; the process of exosome generation is regulated by a variety of factors. Tetraspanin 6 (Tspan6) promotes lysosomal degradation and exosome secretion through the recruitment of syntenin, and proteins such as SNEAR and Rab11, Rab27A/B, and Rab35 promote fusion of MVBs with the plasma membrane to release exosomes. There are several exosome–recipient cell interaction modes. Exosomes can be uptaken and internalized by recipient cells through various endocytosis pathways and deliver bioactive substances to the receptor cells. Exosome membrane proteins can also bind to target cell membrane proteins. Exosomes activate intracellular signaling pathways in the target cell by binding to the recipient cell via the adhesion molecules they carry. Direct membrane fusion is another mechanism for the internalization of exosomes. (By Figdraw. Hangzhou, Duotai. Technology Co., Ltd., Hangzhou 310000, China).

The fusion of MVBs with the plasma membrane initiates exosome secretion and release into the local and distant environment. The secretion of exosomes out of cells mainly relies on the auxiliary effects of the soluble *N*-ethylmaleimide-sensitive factor attachment protein receptor (SNARE) complex and the Rab family proteins (Figure 3).

#### 2.5.1. SNARE Complex and Exosome Release

SNAREs are integral membrane proteins (v-SNAREs on vesicles, t-SNAREs on the target organelles), including VAMP4, STX6, STX16, VTI1A, VAMP3, STX13, etc. [106]. The final step in exosome generation is that MVBs fuse with the plasma membrane (PM). SNARE complex-mediated exosome release is considered to be the classic pathway for the fusion of the fully matured MVBs with the plasma membrane to release exosomes out of the cell [83,107]. As the main components of the SNARE complex mediating membrane fusion, both SNAP25 and VAMP2 can affect the secretion of exosomes [108,109]. Studies on the mechanism of secretory vesicles and membrane fusion in cells such as nerves and pancreatic islets have shown that it is a crucial step that v-SNAREs anchored on the MVB membrane are recruited to t-SNARE on the plasma membranes [110]. At the beginning of the membrane fusion, Rab proteins, Rab effector proteins, and v-SNAREs on the MVBs interact with the target SNARE (t-SNARE) on the inner surface of the plasma membrane to form a specific SNARE complex. The SNARE’s core complex is then assembled by forcing the two membranes to approach and curve. After that, Ca^2+^ combines with synaptotagmin (syt), triggering the SNARE allosteric process. During the allosteric process, t-SNARE and v-SNARE are chimeric together using the action of molecular forces, which pull the two-layer membrane together and finally fuse the vesicle membrane to the plasma membrane. MVB-PM fusion results in MVB-coated intraluminal vesicles release via exocytosis into the extracellular space to become mature exosomes [111]. Increasing evidence has shown that calcium ion-sensitive regulatory proteins involved in membrane fusion events, such as synaptotagmin, play an important role in the regulation of exocytosis of exosomes through Ca^2+^ binding [112]. Studies have shown that the same SNARE proteins (e.g., Ykt6) in different types of cells promote the fusion of MVBs with the plasma membrane, leading to the secretion of different subpopulations of exosomes. Therefore, the inhibition of specific SNARE proteins may change the secretion of only a subset of exosomes [113]. However, the underlying regulatory mechanisms need to be explored by future investigations.

#### 2.5.2. Tethering and Fusion of MVB Vesicles to the Plasma Membrane

Tethering factors regulate the initial contact and establish the connection between transport vesicles and target membranes, promoting the SNARE-mediated membrane fusion process [114]. Currently, studies in yeast and animal cells have identified a series of tethering complexes, among which the exocyst plays a key role in the tethering process of secretory vesicles to the plasma membrane [115,116]. The exocyst is an evolutionarily conserved octamer composed of SEC3, SEC5, SEC6, SEC8, SEC10, SEC15, Exo70, and Exo84 [117].

Exocysts associated with SNARE proteins mediate the downstream membrane fusion events regulated by members of the Ras GTPase protein family, such as Arf6, AP1B, and Rab11 [118]. SEC3 can interact with the small GTPases Cdc42 and Rho1 and participate in the assembly of cytosolic complexes on target membranes [118]. Exo70 can also interact with Cdc42 and Rho3 to mediate the fusion of secretory vesicles and the plasma membrane [103,119]. SEC15 is recruited to secretory vesicles through interaction with secretory vesicle-associated Rab GTPase (SEC4) [120] and also promotes the release of motor proteins after vesicles fuse with the plasma membrane [121]. It has been revealed that exocysts are highly expressed in head and neck cancer tissues. The exocyst interacts with the small GTPase Rab11A that binds to MVBs, mediates the process of Rab11A transporting MVBs to the plasma membrane, and promotes the secretion of exosomes from tumor cells [122].

## 3. Interaction between Exosomes and Recipient Cells

Once released into the extracellular environment, exosomes may conduct physiological and pathological functions through interacting with recipient cells (Figure 3). There are several exosome–recipient cell interaction modes depending on the cell type. Exosomes can be taken up and internalized by recipient cells through various endocytic pathways, as mentioned above, and deliver bioactive substances they carry to the recipient cells. In addition to the endocytic pathway, exosome membrane proteins can bind to recipient cell membrane proteins, thereby activating intracellular signaling pathways in recipient cells. Adhesion to recipient cells and subsequent uptake can also be regulated by the large number of adhesion molecules they carry. Upon binding to cells, exosomes are also internalized by membrane fusion [123]. The processes for the interaction between exosomes and recipient cells are complex and depend on the origin of either exosomes or the recipient cells. Thus, the interaction mode and the mechanisms of exosomes with the recipient cell are not fully understood.

### 3.1. Exosome Membrane Protein–Recipient Cell Membrane Protein Interaction Mediates Signal Transduction

The International Society for Extracellular Vesicles has summarized the marker proteins for demonstrating exosomes in MISEV2018, including tetraspanins (CD37, CD53, CD63, CD81, CD82), major histocompatibility complex (MHC) class I, integrins, transferrin receptor, LAMP (lysosome-associated membrane protein), CD73, CD55, CD86, and so on [124]. It has been reported that exosomal membrane proteins can activate intracellular signaling pathways and affect corresponding recipient cell functions (Table 2) [125]. Metastatic melanoma releases extracellular vesicles, primarily in the form of exosomes, carrying PD-L1 on their surface. Interferon-γ (IFN-γ) stimulation increases the amount of PD-L1 on these vesicles, thereby inhibiting CD8 T-cell function and promoting tumor growth [126]. The exosome membrane secreted by APCs (antigen-presenting cells) contains an antigen-presenting peptide-MHC class I complex, which binds to CD8^+^ T cells, activates the proliferation and differentiation of T cells, and induces the body to produce an immune response [127]. It has been shown that exosomes from dendritic cells (DCs) carry major histocompatibility complexes (MHCs) I and II and thus interact with T cells to elicit an immune response. DC-derived exosomes carrying the T-cell co-stimulatory molecule CD81 cognize T-cell receptors to activate T cells [128]. Exosomes carrying MHC II molecules can be internalized by DC cells for translocation to the DC surface and presentation to CD4^+^ T cells [129,130]. CD37 is selectively expressed on the surface of mature B cells and exosomal membranes. Recent studies have shown that CD37 participates in both pro-survival and pro-apoptotic signaling through the PI3K/AKT pathway. In addition, it controls IL-6 receptor signaling through its interaction with SOCS3. Furthermore, CD37 can mediate the aggregation of α4β1 and subsequent activation of PI3K/AKT signaling and cell survival [131,132]. CD63 may interact with the integrins α4β1, α3β1, α6β1, LFA-1, and β2 [133], mediating binding to the ECM (extracellular matrix) and promoting tumor cell migration. However, the mechanism of this process is unclear. It has been suggested that CD63 is able to interact with other tetratransmembrane proteins, thus affecting the activity and stability of integrins [134]. Stress-induced heat shock proteins (HSPs) are endogenous “danger signals” that increase tumor immunogenicity and induce natural killer (NK) cell responses. Exosomes are a novel secretory pathway for heat shock proteins. Li et al. showed that exosomes derived from hepatocellular carcinoma cell stimulated by anticancer drugs carried more HSP60, HSP70, and HSP90, and that these HSPs directly triggered NK cell activation, promoting in vitro migration and cytotoxicity to hepatocellular carcinoma cells [135,136].

### 3.2. The Regulation of the Adhesion Molecule in Exosome–Recipient Cell Interactions

Integrins and adhesion molecules presenting on the surface of exosomes also mediate interactions with target cells, attachment, and membrane fusion. Hoshino et al. [137] analyzed the proteomes of exosomes from several kinds of tumors, including breast cancer, found that the integrins α6β4, α6β1, and αvβ5 were enriched in exosomes, and applied them to stromal cells, lung macrophages, and liver Kupffer cells to determine the effects of exosome on target cells. The results showed that integrin-enriched exosomes could promote Src activation and expression of the pro-inflammatory factor S100, which promotes tumor metastasis. Therefore, in addition to adhesive properties, exosomal integrins could activate Src and upregulate pro-migratory and pro-inflammatory molecules in specific resident cells in the microenvironment of distant tissues, thereby promoting lung and liver metastasis of breast cancer cells [137]. Hoshino et al. [137]. found that adhesion molecules on the membrane of exosomes determine the interaction of exosomes to specific cell types and organ-specific ECM molecules. For example, exosomes with ITGαvβ5 bind to liver Kupffer cells, while exosomes containing ITGα6β4 and ITGα6β1 bind to lung-resident fibroblasts and epithelial cells (Table 2) [137]. Exosomes from cancer cells interact with the cell surface uptake receptor, heparan sulfate proteoglycans (HSPG), to mediate exosome uptake and functional activity [138]. Members of the lectin family have been shown to play a role in exosome internalization. Lectin family members have been identified on various cell membranes as well as exosomal membranes. For example, C-type lectin receptors have been found on dendritic cells and brain endothelial cells and interact with C-type lectins to internalize macrophage-derived exosomes [139]. The macrophage receptor with collagen structure (MARCO) is a scavenger receptor alpha-like protein expressed on the surface of macrophages. It has been shown that MARCO can not only mediate the binding and uptake of environmental particles, including nanomaterials, but also regulate the internalization of exosomes. These results suggest a role for MARCO in exosome uptake [140].

### 3.3. Direct Membrane Fusion of Exosomes with Plasma Membranes of Recipient Cells

Direct membrane fusion is another mechanism for the internalization of exosomes, through which the membrane of exosomes directly merges with the plasma membrane and injects cargo molecules into the recipient cells [141]. For example, the lipid bilayers of exosomes derived from dendritic cells can approach the plasma membrane of recipient cells in an aqueous environment and make the outer leaflets contact each other directly, leading to the formation of a hemifusion stalk, and finally, a fusion pore opens, which releases contents of the exosomes into the recipient cell [142,143]. Previous studies have shown that a range of proteins, such as SNAREs, Rab proteins, SM proteins (Sec1/Munc-18-related proteins), and LAMP-1, are involved in this process of direct membrane fusion [143]. The membrane fusion process is also influenced by various factors, such as the microenvironment and membrane composition. For example, acidic conditions promoted the fusion of exosomes derived from melanoma cells with the plasma membrane of recipient cells. However, a change in membrane composition by using filipin treatment to associate cholesterol inhibited the membrane fusion activity [113,144].

## 4. Regulation of Exosome Production in Cancer Cells

Compared with normal cells, cancer cell-derived exosomes have differences in number, content, and function. Although the exact mechanism is not fully unraveled, it has been suggested that dysregulation of exosome production in cancer cells may be the result of the activation of oncogenic signals or altered microenvironmental conditions. The activation of oncogenes and inactivation or loss of tumor suppressor genes are key events in cancer progression, and several oncogenes and tumor suppressors have been found to be closely related to the regulation of exosome secretion.

### 4.1. Regulation of Exosome Formation Mediated by Ras

*Ras* is one of the most prominent oncogenes in many cancer cell types and is predominantly found in colon, pancreatic, and lung cancers [145]. Mutations in the *ras* gene are the most common mutations in human tumors, and the Ras signal directly regulates exosome secretion and sorting of exosome cargos [146]. Studies have shown that exosomes from mouse glioblastoma (GBM) cells are enriched in active Ras and that Ras also co-precipitates with the ESCRT-associated exosomal proteins Vps4a and Alix in lysates of EVs derived from both human and mouse GBM cells, suggesting that Ras is involved in exosome sorting [146]. Ras-transformed mouse fibroblasts NIH3T3 secreted exosomes have 34 more proteins than untransformed fibroblasts exosomes, of which lactoferrin, collagen α-1 (VI), 14-3-3 isoforms, guanine nucleotide-binding proteins (G proteins), eukaryotic translation initiation factors elF-3γ and elF-5A accumulate in exosomes of cells following Ras-induced oncogenic transformation, in contrast to *ras* mutations that reduce the sorting of specific miRNAs into exosomes [147]. Furthermore, in colorectal cancer cells, Ras-MEK signaling phosphorylates the RNA-binding protein Ago2, which reduces the exosome secretion of Ago2 and Ago2-bound miRNAs [148]. Based on the existing findings, Ras plays an important role in regulating exosome secretion and sorting of exosome cargos. However, the mechanism underlying the link between Ras activity and exosome formation remains to be further identified.

### 4.2. Regulation of Exosome Formation Mediated by Rab Proteins

Rab proteins, like Ras proteins, are members of the small GTPase family and are highly conserved regulators of vesicle transport. As mentioned previously (Section 2.4.2), Rab proteins are important molecular switch proteins in the process of vesicle formation, transport, anchoring, and fusion and play a very important regulatory role in all aspects of vesicle transport [91]. Rab proteins also play a critical role in exosome production in cancer cells, and changes in Rab expression or activity interfere with exosome release, thereby affecting cancer progression [149]. For example, reduced activity of Rab11 in human chronic myeloid leukemia cells (K562) reduces exosome release, and Rab11 co-localizes with LC3 during autophagy, which may be associated with reduced exosome release [150]. Furthermore, the knockdown of Rab27A in an invasive cancer cell line reduced exosome secretion, whereas Rab27A-mediated exocytosis increased cell migration, chemotaxis, and invasion [151]. Using an RNA interference (RNAi) screen, Ostrowski et al. [152] identified five Rab GTPases that promoted exosome secretion in HeLa cells, including Rab2B, Rab9A, Rab5A, Rab27A, and Rab27B. Knockdown of these five Rab proteins inhibits exosome secretion without significant modification of soluble protein secretion via the conventional secretory pathway. Among them, Rab27A and Rab27 B perform distinct and non-redundant tasks in the intracellular transport of MVEs. Rab27A and Rab27B can promote exosome release by facilitating the targeting of MVEs to the periphery of the cell and docking them at the plasma membrane. Silencing Rab27A promotes the occurrence of fusion between MVEs, whereas Rab27B silencing causes MVEs to be redistributed to the perinuclear region and inhibits the release of MVEs [152]. Slp4 and Slac2b are effectors of Rab27A and Rab27B, respectively, and in HeLa cells, Slp4 interacts preferentially over Rab27B with Rab27A to regulate exosome secretion [153]. Currently, studies on the regulation of exosome secretion by Rab11 and Rab27A/B have mainly focused on in vitro cellular experiments, and whether they have similar functions in vivo remains to be further explored.

### 4.3. The Modulation of Exosome Biogenesis by the Epidermal Growth Factor Receptor

Epidermal growth factor receptor (EGFR or ErbB-1 or HER1) is encoded by the proto-oncogene *c-erbB-1*. Recent studies have revealed that constitutively active EGFR mutants can drive their own secretion by promoting the production of Rab31-dependent exosomes. Active EGFR phosphorylates Rab31 in late endosomes, leading to activating TBC1D2B protein and inactivating Rab7, which prevents MVBs from fusing with lysosomes and drives raftin (flotillin)-dependent ILVs formation [7]. Furthermore, it should be noted that exosomes derived from cancer cells with EGFR mutations exhibit distinct cargo-sorting patterns. For example, EGFRL858R-activating mutations have the ability to modify the protein composition in non-small-cell lung cancer cells and glioblastoma cells. The exosomes derived from cells with the EGFRL858R mutation show a higher concentration of proteins involved in the E2F and MYC signaling pathways [154]. Exosomes derived from cells transformed by EGFRvIII have a higher concentration of proteins linked with adhesion plaques and invasion-related proteins, such as CD44, BSG, and CD151 [155].

### 4.4. The Control of Exosome Biogenesis Mediated by c-Myc

The oncogene *c-myc* is a member of the *myc* gene family. The *c-myc* gene has been associated with the development of many tumors. Recent studies have found that the MEK/ERK/c-MYC pathway prevents MVBs degradation and promotes tumor-derived exosome biogenesis by inhibiting lysosomal function [156]. In contrast, inhibition of the MEK/ERK pathway results in nuclear translocation of the MiT/TFE transcription factor and suppression of the expression of *c-myc*. This promotes the transcription activation of lysosome-associated genes, including those that encode the subunits of the vesicular-type H^+^-ATPase, which in turn facilitates acidification, activating the function of lysosomes, and inhibits the release of EVs [156]. In the context of renal cancer cells and tissues stimulated by MEK/ERK, the overexpression of c-MYC was found to be correlated with the downregulation of genes relevant to lysosome activity [156]. This finding indicates that in cases of human malignancies, the MEK/ERK/c-MYC pathway plays a crucial role in regulating the fate of MVBs and facilitating the production of EVs by impairing the function of lysosomes.

### 4.5. The Regulation of Exosome Biogenesis Mediated by Src

The Src protein, a kind of tyrosine kinase, assumes an essential function in cellular signaling pathways that control cell growth, adhesion, and migration. Consequently, the activation of Src contributes to the promotion of cancer occurrence and progression. During the initial stages of cancer development, c-Src is activated and transduces carcinogenic signals [157]. Hikita et al. [157] found that interactions between the endosome membrane-anchoring c-Src SH3 domain and the proline-rich region of Alix enhanced the ESCRT-mediated synthesis of ILVs following a rise in exosome secretion in c-Src transformed cells. Moreover, the activation of c-Src kinase facilitates the release of synaptic vesicle-associated protein 23 (SNAP23). SNAP23, a crucial t-SNARE, plays a significant role in the promotion of SNARE complex assembly via its phosphorylation. This process leads to the reduction of cholesterol and the generation of ILVs, hence facilitating an increase in exosome secretion. It has also been demonstrated that the SNAP23-cholesterol pathway in pancreatic cancer cells promotes the secretion of EVs [158].

### 4.6. The Modulation of Exosome Biogenesis Mediated by Syntenin

Syntenin has been identified as an oncogene that participates in multiple signaling pathways. With the PSD-95, Dlg, and ZO-1 (PDZ) domains, syntenin, as an adaptor protein and scaffold protein, is involved in cellular physiological regulation. Syntenin promotes cancer development, metastasis, and angiogenesis in various cancers [159]. The protein Syntenin-1 has been found to be involved in the biogenesis and release of exosomes [159]. It has been observed that Syntenin-1 facilitates the formation of CD63-bearing exosomes by recruiting Alix and ESCRT proteins to endosomes [160].

### 4.7. The Control of Exosome Biogenesis Mediated by p53

Oncogenes are known to have a significant impact on the regulation of tumor exosome production. Currently, *p53* is thought to be the most significant oncogene, and *p53* mutations are found in more than 50% of cancers [161]. It is suggested that missense *p53* mutants, aside from their various intracellular activities that facilitate tumor progression, also exert an influence on intercellular communication within the tumor microenvironment. Specifically, these mutants impact the secretion of exosomes by cancer cells, thereby altering their composition and quantity. For instance, it has been observed that human colon cancer cells with *p53* mutant exhibit a specific release of exosomes that are abundant in miR-1246. These exosomes play a role in facilitating the miR-1246-dependent reprogramming of macrophages under the microenvironment of tumors. Following reprogramming, tumor-associated macrophages limit the anti-inflammatory effects and enhance the activity of TGF-β, facilitating the advancement of tumors [162]. Ju et al. [163] observed that the presence of *p53* mutations led to an elevation in the quantities of miR-21-3p and miR-769-3p in exosomes. Furthermore, the authors noted that both miR-21-3p and miR-769-3p exhibited a capacity to activate fibroblasts. Fibroblasts that have been activated are able to secrete the cytokine TGF-β, which in turn can initiate the process of epithelial-mesenchymal transition (EMT) in cancer cells [159]. Nevertheless, the exact mechanism by which *p53* regulates exosome secretion remains unclear.

### 4.8. The Regulation of Exosome Biogenesis Mediated by Jagged-1

Jagged-1 (JAG1) is one of the typical ligands of the Notch signaling pathway, which is involved in vascular sprouting and is a poor prognostic factor for TNBC (triple-negative breast cancer) [164]. The Notch ligands, JAG1 and DLL4, are key factors affecting the interaction of tumor cells with their neighbors and are involved in angiogenesis [164]. JAG1 can not only participate in the growth, proliferation, and migration of tumor cells but can also promote the formation of treatment tolerance. Recent studies have found that Notch receptors or ligands can be loaded into exosomes to exert long-distance effects and even influence exosome secretion [165]. It was shown that JAG1 was highly expressed in MDA-MB-231 bone (231B) cells, and its invasion and metastatic ability was stronger than that of MDA-MB-231 (231) cells, and JAG1 was able to upregulate the protein levels of Alix, as well as Rab11A and Rab35 to promote exosome secretion in MDA-MB-231 bone (231B) cells. At the same time, JAG1 upregulation also increased the level of MALAT1-miR-140-5p in exosomes, which promoted angiogenesis in TNBC [166].

## 5. The Tumor Microenvironment Regulates the Biogenesis and Function of Exosomes

Tumor cell-derived exosomes (TDEs) regulate the tumor microenvironment (TME) in an autocrine or paracrine manner to make it conducive to their own progression [167]. The characteristics of the tumor microenvironment mainly include overall hypoxia, acidification, interstitial hypertension, vascular hyperpermeability, inflammatory reactivity, and immune suppression. Apart from cell-autonomous mechanisms, tumor microenvironmental conditions also help regulate exosome release from tumor cells [168]. Hypoxia is a significant feature of the tumor microenvironment, and acidification is an indirect result of hypoxia as well. Hypoxia may affect key regulatory nodes of exosome formation and secretion, such as exosome cargo sorting, MVB transport, and fusion with the plasma membrane and release [168].

HIF (hypoxia-inducible factor) is the main component of the hypoxia-related signaling pathway and contains multiple subunits such as HIF-1α or HIF-2α and HIF-1β/ARNT. Under hypoxic conditions, HIF is activated and participates in the regulation of cellular oxygen homeostasis, red blood cell production, oxygen consumption, angiogenesis, mitochondrial metabolism, and exosome secretion and composition [169]. A study on hypoxic exosome production in rat renal proximal tubule cells (RPTCs) showed that HIF-1 extremely significantly increased exosome production in a time-dependent manner. But it does not change the average size of exosomes secreted by RPTC [170]. Further analysis found that under hypoxic conditions, HIF regulates exosome secretion by activating Rab22A [171]. Rab22A localizes to early endosomes and is required for sorting and recycling of molecular cargos internalized by clathrin-dependent and -independent mechanisms. Rab22A is closely related to Rab5 and shares effectors with Rab5 and Rab4 [99,100]. Rab22A has been shown to be overexpressed in a variety of tumors [172,173]. Rab22A overexpression significantly affects the morphology and function of early endosomes, thereby affecting the sorting and recruitment of cargo proteins [174]. In addition, the study of Dorayappan et al. showed that hypoxia could increase the release of exosomes by upregulating Rab27A and reducing Rab7, LAMP1/2, and NEU-1 [175]. Hypoxia can also alter exosome secretion by affecting intracellular transport. Studies have shown that under hypoxic conditions, the actin cytoskeleton, microtubules, and molecular motors will change in cells, thereby promoting cancer cell invasiveness. Inflammatory reactivity is another major characteristic of the tumor microenvironment. Inflammatory stress can change the composition and secretion of exosome-derived tumors. Olivier G et al. [176] found that when endothelial cells are exposed to stress conditions such as hypoxia and TNF-α-induced inflammatory activation, the protein and mRNA abundance in endothelial cell-derived exosomes changes. A study on exosome secretion from human keratinocytes found that induction of hypoxia and H_2_O_2_ (which induces inflammatory stress response) can make a variety of normal and tumor cell responses more dependent on the transforming growth factor-alpha (TGF-α) non-canonical pathway. The activation of TGF-α/Akt/PRAS40, in turn, affects exosome secretion [177].

Acidification of the cancer cell microenvironment can also influence exosome formation or absorption. For example, low pH in the microenvironment will affect integrin activity and membrane composition of the cell membrane and will, thus, increase exosome release and enhance the uptake of exosomes into recipient cells [178,179]. Nutritional deficiency and dysregulated protein synthesis in cancer cells leads to increased protein misfolding and endoplasmic reticulum (ER) stress. ER stress increases MVB formation and subsequent exosome release via the ER stress sensors inositol-requiring enzyme 1 (IRE1) and PKR-like ER kinase (PERK). In choriocarcinoma cells, severe endoplasmic reticulum stress is associated with the secretion of exosomes containing DAMP molecules. Endoplasmic reticulum stress also triggers the splicing of X-box binding protein 1 (XBP1), increasing its content in exosomes [180]. Calcium signaling is related to exosome secretion, assembly, and uptake. Studies have shown that Munc13-4 is upregulated in invasive cancer cells and is involved in the maturation of MVBs, and the increase in Munc13-4 is related to calcium uptake [181].

## 6. Outlook and Conclusions

Cancer cells release more exosomes than healthy cells, suggesting a potential relationship between tumors and exosomes. Exosomes regulate the functions of tumors and/or host cells and promote tumor occurrence, progression, and metastasis by delivering the proteins, DNA, mRNA, and miRNA they carry. At the same time, exosomes are closely related to the occurrence of tumor drug resistance. Tumor cells can protect themselves from the cytotoxic effects in the immune system by increasing the efflux of chemotherapy drugs, thereby transmitting drug-resistant miRNA, enhancing autophagy, and inhibiting apoptosis, etc.

In addition, the uniqueness of exosomes provides them with a special potential in disease diagnosis and treatment. Proteins located on the surface or within the membrane of exosomes can also be used as cancer biomarkers. Exosomal proteins have been reported as specific diagnostic and prognostic factors for a variety of cancers, including breast cancer, lung cancer, gastric cancer, and liver cancer [182]. At the same time, exosomes can be engineered to contain desired proteins to prevent tumor progression, potentially being used in cancer treatment. The immune functions of exosomes may enable their use as specific drug delivery vehicles or vaccines for cancer immunotherapy.

It can be seen that exosomes have become a hot research topic in the field of life sciences. Despite this, the mechanisms of exosome biogenesis and secretion are still not fully understood. In particular, there is a lack of clear understanding of the regulatory mechanisms for increased biogenesis of cancer cell-derived exosomes and changes in cargo composition. Further research is still required on the biogenesis regulation of tumor-derived exosomes in different cancer types and different stages of the same cancer type. Tumor-derived or tumor-related exosomes are important mechanisms that regulate the occurrence and development of tumors. Elucidating these mechanisms will help clarify precise targets and exact pathways for disease intervention. The special synthesis pathways and complex sources of exosomes determine that we still have a long way to go to understand and utilize exosomes, but these are issues worth exploring in depth.

## Figures and Tables

**Table 2:** Interaction of exosome membrane proteins and recipient cells.

Exosome Membrane Proteins	Recipient Cells	References
PD-L1	CD8 T Cell	[126]
MHC I, MHC II, CD81	T Cells	[127,128]
MHC II	Dendritic cells	[129,130]
HSP20, HSP60, HSP70, HSP90	NK cells	[135,136]
αVβ5	Kupffer cells	[137]
α6β4, α6β1	Lung fibroblast cells, epithelial cells	[137]
